# Lifelong Sequelae of a Childhood Tibial Fracture: Severe Procurvatum Tibial Malunion Leading to Secondary Knee Osteoarthritis

**DOI:** 10.7759/cureus.107833

**Published:** 2026-04-27

**Authors:** Ethan Burton, William Haller

**Affiliations:** 1 College of Osteopathic Medicine, Edward Via College of Osteopathic Medicine, Auburn, USA; 2 Department of Orthopedics, Gadsden Regional Medical Center, Gadsden, USA

**Keywords:** knee osteoarthritis, postural compensation, secondary intention fracture healing, surgical secondary knee osteoarthritis management, tibial malunion

## Abstract

The sequelae of tibial malunion following surgical repair in the pediatric population are well documented. Persistent tibial malunion introduces varus or valgus forces that predispose patients to knee compartment overload, limiting ambulation and promoting osteoarthritis and osteoporosis. To prevent these sequelae, tibial malunions are categorized by deformity severity to guide surgical interventions, including intra-articular, extra-articular, and combined approaches. A male in his early 70s presented to the orthopedic clinic for reevaluation of severe right knee osteoarthritis secondary to a longstanding tibial malunion healed in malalignment after failed fixation and the absence of revision surgery. Radiographs demonstrated a 57.2-degree procurvatum and 30.9-degree valgus deformity of the tibia. Initial management included multiple courses of intra-articular corticosteroid injections that provided minimal relief, with progressively worsening ambulation due to knee pain. A total knee arthroplasty was performed. Intraoperative challenges included poor bone quality and retained tibial hardware, limiting stem length and placement. At three months postoperatively, the patient reported significant improvement in ambulatory capacity and knee pain despite the persistence of the extra-articular deformity. There are limited reports of severe tibial malunions persisting into the geriatric period, as well as their long-term biomechanical consequences. Furthermore, there are limited reports describing total knee arthroplasty performed without correction of extra-articular deformity. This case suggests that, in select geriatric patients, extra-articular tibial deformities may be managed with isolated total knee arthroplasty when osteotomy is high-risk or undesirable. However, the findings are constrained by the single-case design, limited follow-up duration, and absence of objective postoperative alignment data.

## Introduction

Tibia and fibula fractures are common injuries, with the Swedish Fracture Register showing an incidence of 51.7 per 100,000 person-years, with a higher prevalence in males [1]. Currently, there is limited epidemiologic data regarding tibial fracture incidence in the United States. Tibial malunion is a well-documented complication, with studies in the United States reporting malunion rates of up to 23.8% following intramedullary nail fixation [2]. Furthermore, isolated case reports have described patients who were lost to follow-up after initial fixation and later presented with failed fixation and subsequent malunion [3-5].

The link between tibial malunion and osteoarthritis is well established [6]. Valgus and varus forces overload the lateral and medial knee compartments, respectively. The increased mechanical load on each knee compartment contributes to the development and progression of osteoarthritis. Cohort studies have demonstrated that patients with valgus and varus forces on the knee joint as a result of trauma exhibit more severe signs and symptoms of osteoarthritis [7]. 

The known link between mechanical axis deviation and compartment loading has led to tiered management strategies for osteoarthritis secondary to tibial malunion. The recommended current interventions include total knee arthroplasty, osteotomy, or a combined approach [8,9]. The treatment approach is generally chosen based on the severity of the deformity. Newer strategies for management also include personalized arthroplasties that attempt to correct axis deviation intra-articularly while leaving the extra-articular deformity [10]. 

In this case, lateral radiographs demonstrated a procurvatum deformity, anterior angulation of the tibia, measuring 57.2 degrees, while coronal imaging revealed a valgus deformity, lateral deviation of the distal tibia from the midline, measuring 30.9 degrees. Deformities exceeding ≥10 degrees in the sagittal plane or ≥5 degrees in the coronal plane are generally considered unacceptable and often warrant corrective intervention [11]. However, few reports describe outcomes in patients with severe tibial malunion and secondary osteoarthritis managed with isolated total knee arthroplasty.

## Case presentation

A male in his early 70s presented with chronic right knee pain and progressive limitation in ambulation. He reported a long-standing deformity of the right lower extremity dating back to a childhood injury.

The patient sustained fractures of the right tibia and fibula during his teenage years. Initial management involved surgical fixation of the tibia using screws and cerclage wire. The initial fixation failed, resulting in progressive deformity. No revision surgery was attempted at the time due to the patient's preference.

The patient reported right lower extremity pain for more than four decades, with progressive knee pain over the preceding five years. Knee symptoms had been managed medically with acetaminophen, nonsteroidal anti-inflammatory drugs, and corticosteroid injections prior to presentation. He reported progressive functional limitations, including reliance on a walker for ambulation. Mobility was further impaired by a leg length discrepancy and deformity-related mechanical inefficiency. The patient’s BMI was 31.7 kg/m^2^, and he was a former smoker.

Past medical history was significant for chronic pain of multiple joints, hypertension, hyperlipidemia, right quadriceps muscle weakness, and osteoarthritis of the left hip. Past surgical history was significant for left hip arthroplasty with revision and a remote left femur fracture with limb shortening.

**Figure 1:**
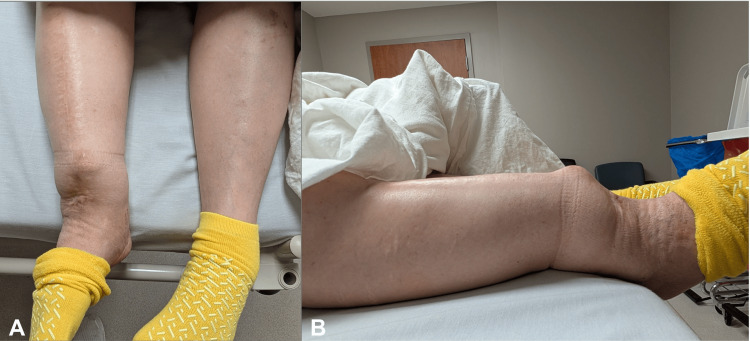
Gross view of the lower extremities (A) Anteroposterior view of the right lower extremity demonstrating deformity. (B) Lateral view demonstrating a pronounced procurvatum deformity of the tibia.

Examination revealed a markedly deformed right lower extremity with a visible procurvatum tibial deformity (Figure 1). The proximal tibia was anterior to the distal shaft, and the affected limb was shortened by approximately one centimeter, as obtained by comparing the medial malleolus. Knee and ankle range of motion were limited. Lateral joint line tenderness was greater than medial tenderness. No ligamentous instability was appreciated. Neurovascular examination of the right lower extremity was intact. 

The patient’s presentation reflects a combination of symptoms attributable to both knee osteoarthritis and leg length discrepancy. Osteoarthritis-related features included limited knee range of motion, pain, and joint line tenderness. In contrast, leg length discrepancy contributed to altered gait mechanics and secondary changes in spinal and hip alignment, resulting in chronic hip and back pain.

**Figure 2:**
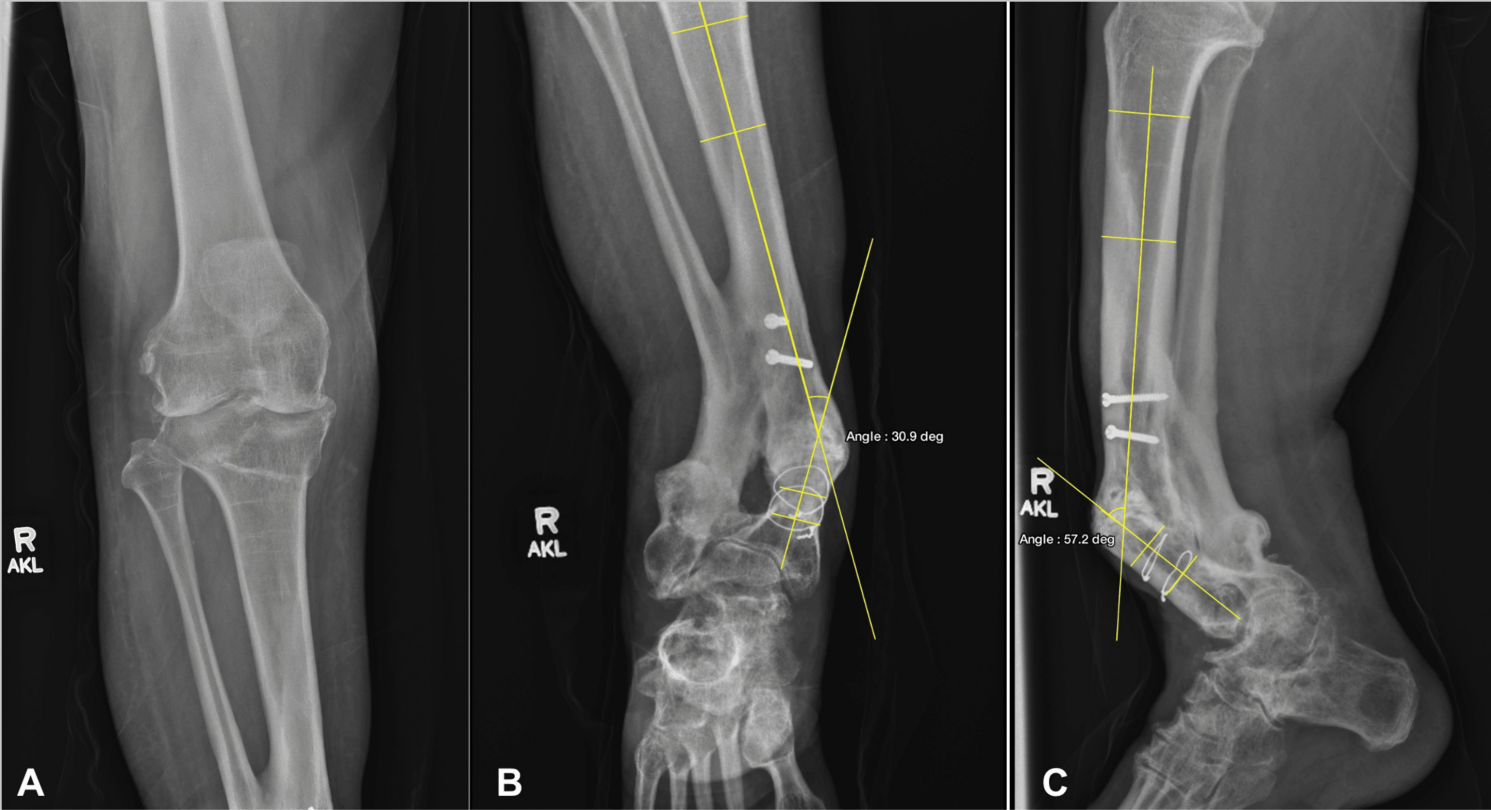
Labeled radiographs of the right knee and lower leg (A) Anteroposterior radiograph of the right knee demonstrating joint space narrowing in both compartments with associated osteophyte formation. (B) Anteroposterior radiograph demonstrating 30.9° valgus deformity of the right tibia with ossification of the interosseous membrane and retained proximal tibial screws and cerclage wires distally. (C) Lateral radiograph demonstrating 57.2° procurvatum deformity. Angles were measured using lines drawn along the anatomical axes of the proximal and distal tibial segments, with the intersection representing the degree of deformity in the coronal and sagittal planes.

Anteroposterior knee radiographs demonstrated advanced, predominantly lateral compartment osteoarthritis with osteophyte formation, consistent with chronic mechanical axis deviation from an extra-articular tibial deformity. Tibial imaging revealed a 30.9° valgus deformity and a 57.2° procurvatum malunion, with associated interosseous membrane ossification and retained hardware (Figure 2).

The patient was diagnosed with severe right tibial malunion secondary to a childhood fracture, resulting in knee osteoarthritis from long-standing altered mechanical axis of the knee.

Multiple surgical options were discussed with the patient. Deformity correction was not pursued due to the chronic nature of the malunion and the extensive surgical burden. The patient expressed a preference for amputation rather than deformity correction should arthroplasty fail to provide symptomatic relief.

A cemented total knee arthroplasty was performed using a Zimmer Biomet Persona system (Zimmer Biomet, Warsaw, IN, USA), including a size 9 cruciate-retaining femoral component, a size E revision tibial tray with a 14 × 75 mm stem extension, a 10 mm medial congruent polyethylene insert, and a size 38 patellar component. Computer-assisted imageless navigation was used to guide alignment. A stemmed tibial component and cement fixation were used.

**Figure 3:**
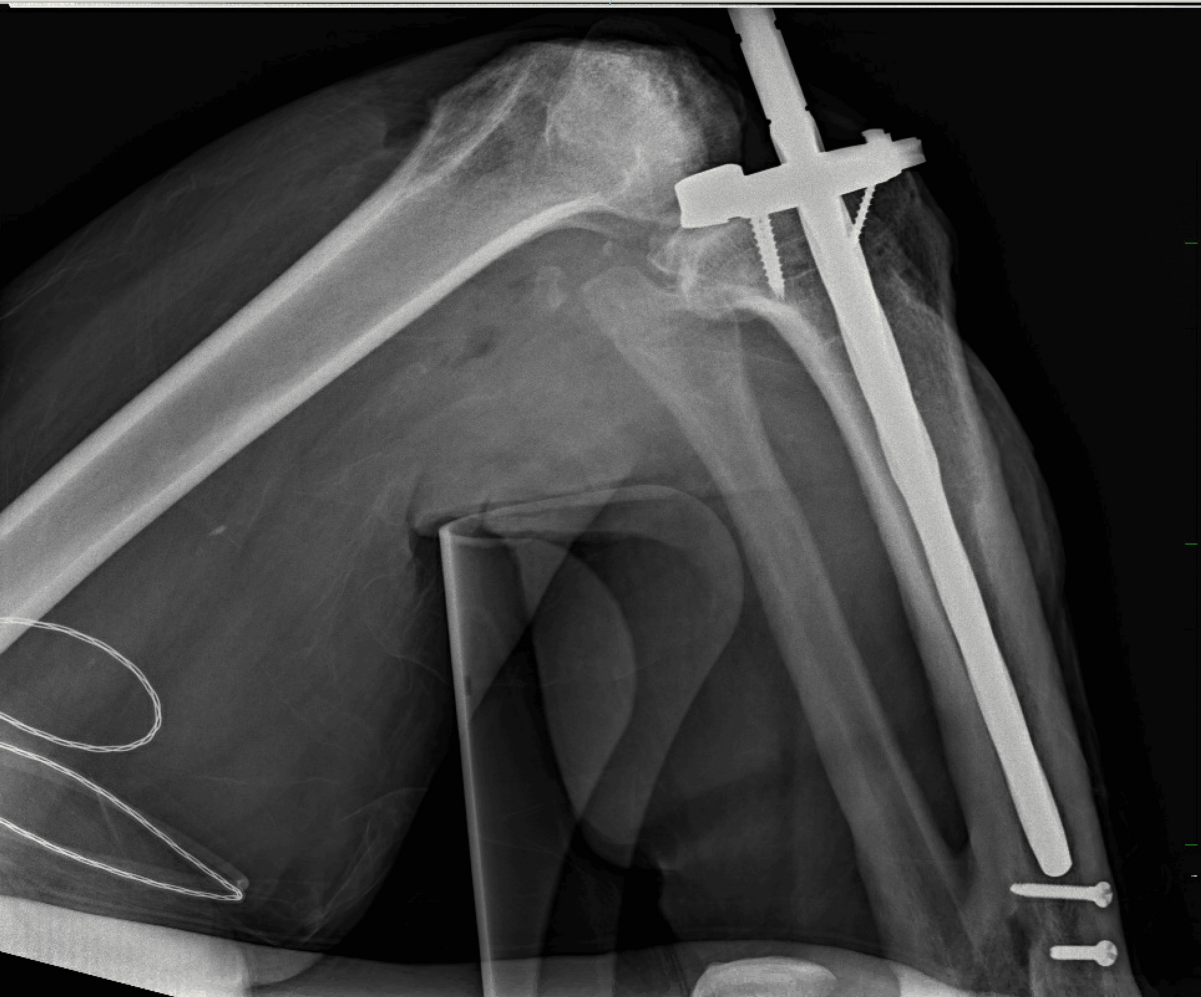
Intraoperative lateral radiograph of the right leg Lateral intraoperative radiograph demonstrating tibial component and stem placement. Proximity of the tibial stem to retained tibial screws demonstrates intraoperative stem length constraints. Increased lucency of the proximal tibial cortical and medullary bone suggests decreased bone density.

Intraoperative challenges included poor medullary quality and retained tibial hardware, limiting stem length and placement (Figure 3). Polymethylmethacrylate cement augmentation was used on the tibial component and stem to ensure stable fixation and alignment. 

Three months postoperatively, the patient reported significantly improved ambulatory capacity and reduced pain. 

## Discussion

**Figure 4:**
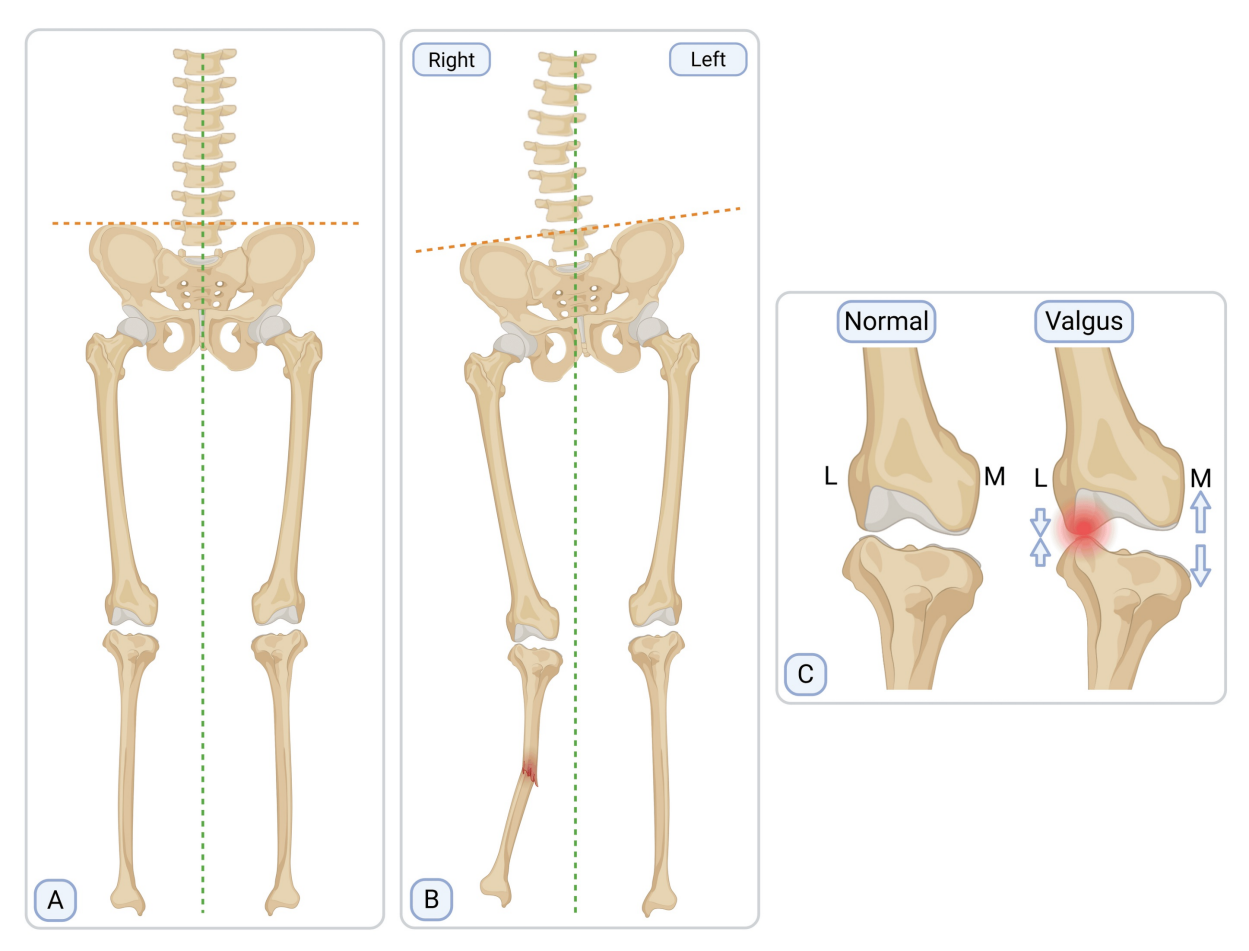
Postural compensation (A) Demonstrates normal midline spinal alignment (green dashed line) and level iliac crests (orange dashed line). (B) Demonstrates alignment changes associated with limb shortening secondary to valgus tibial deformity on the right, including ipsilateral pelvic tilt and functional lumbar scoliosis. (C) Arrows demonstrate narrowing of the lateral (L) knee compartment and widening of the medial (M) knee compartment with a valgus tibial deformity. Figure created by the author using BioRender.com (Burton, E. (2026) https://BioRender.com/dfksvxr)

Tibial malunion is classified as acceptable or unacceptable for the purposes of surgical candidacy. Unacceptable malunions are defined as ≥10 degrees in the sagittal plane or ≥5 degrees in the coronal plane. Acceptable malunions are defined as <10 degrees in the sagittal plane and <5 degrees in the coronal plane, with <1 cm of limb shortening [11]. Tibial malunions with leg length discrepancies >2 cm are associated with knee and hip osteoarthritis, low back pain, and functional scoliosis [12,13]. Skeletal complications result from leg length discrepancy, inducing pelvic tilt. Pelvic tilt results in increased stress on the hip joint, leading to osteoarthritis and functional scoliosis to maintain head and trunk position over the pelvis (Figure 4) [14]. Thus, unacceptable deformities are typically managed with corrective osteotomy. This patient’s deformity exceeded accepted thresholds in both planes, meeting criteria for an unacceptable malunion.

Although unacceptable tibial malunions with comorbid osteoarthritis of the knee are typically managed with a combined extra-articular and intra-articular correction, three factors drove the choice for intra-articular correction alone: patient preference, duration of malunion, and age. The chronic nature of the malunion resulted in the degeneration of multiple joints. Furthermore, symptoms due to leg length discrepancy <1 cm often improve with orthotics [15]. The patient’s age also played a role in the decision to avoid corrective osteotomy. Corrective osteotomies for severe tibial malunions are often associated with longer recovery times [16-18]. In geriatric patients, this can be a relative contraindication due to limited life expectancy. In addition, studies have shown that staged procedures increase perioperative risk and recovery burden in elderly patients [19]. Finally, the patient’s suspected poor bone quality due to age and limited ambulation indicated that a corrective osteotomy would be at increased risk for nonunion [20-22]. However, limitations of intra-articular correction alone were discussed with the patient, including the persistence of abnormal mechanical loading across the knee and adjacent joints, which may contribute to ongoing degeneration and could negatively impact long-term implant survivorship.

Instability of the tibial stem after placement and fragile medullary bone were suggestive of poor bone quality, although the diagnosis of osteoporosis was not confirmed. Due to the patient's age and history of limited ambulation, this was an anticipated complication [23]. The length of the tibial component stem was maximized to distribute the mechanical load across the greatest area of medullary bone; however, the length was still limited by retained tibial screws. Studies have shown that increasing tibial component stem length in patients with poor bone quality improves postoperative alignment [24,25].

This report is limited by its single-patient design and relatively short follow-up. Objective baseline and postoperative measures, including validated pain and functional scores, were not consistently available, which limits quantitative assessment of outcomes. Additionally, the patient’s overall ambulatory status may have been influenced by comorbid musculoskeletal conditions, making it difficult to attribute improvement solely to the knee intervention. Despite these limitations, this case provides practical insight into the management of complex tibial malunion in a patient where standard corrective strategies may be high-risk or undesirable.

## Conclusions

This case demonstrates subjective functional improvement in a patient with osteoarthritis following total knee arthroplasty while retaining the extra-articular tibial deformity. These findings suggest that meaningful short-term relief may be achieved with intra-articular correction alone when osteotomy is considered undesirable. Selection of this patient required consideration of deformity severity, patient preference, and comorbid conditions, including age and retained hardware. However, long-term implant survival and the effects of asymmetric joint loading have not been elucidated. Additionally, the interpretation of these findings is limited by the brief follow-up period (three months) and the lack of objective pain and functional outcome measures.
